# Therapeutic Benefits of Robotics and Exoskeletons for Gait and Postural Balance Among Children and Adolescents with Cerebral Palsy: An Overview of Systematic Reviews

**DOI:** 10.3390/healthcare13233120

**Published:** 2025-12-01

**Authors:** Amal Alharbi, Shouq S. Alhosaini, Shahad S. Alrakebeh, Saleh M. Aloraini

**Affiliations:** 1 Department of Physical Therapy, College of Applied Medical Sciences, Qassim University, Buraydah 51452, Qassim, Saudi Arabia; saloraini@qu.edu.sa; 2 Hayat National Hospital, Unayzah 56243, Qassim, Saudi Arabia; dpt.shouq@gmail.com (S.S.A.); pt.shahadsaleh@gmail.com (S.S.A.)

**Keywords:** systematic reviews, robotics, exoskeletons, gait, postural balance, cerebral palsy

## Abstract

**Background/Objectives:** Robotic therapies are emerging as a potential management strategy for individuals with cerebral palsy (CP). These devices apply mechanical and electrical forces to regulate neural excitability and promote motor learning. This review aimed to systematically assess and synthesize evidence from published systematic reviews and meta-analyses on the therapeutic benefits of robotics and exoskeletons for gait and postural balance in pediatric CP. **Methods:** A comprehensive search of PubMed, CINAHL, Scopus, and The Cochrane Library was conducted. Two independent reviewers screened records to identify studies that were: (1) written in English and published in peer-reviewed journals; (2) included participants <18 years with a diagnosis of CP; and (3) examined robotic therapies or exoskeletons targeting gait or postural balance. Methodological quality of included reviews was appraised with the Assessment of Multiple Systematic Reviews (AMSTAR) tool, and certainty of evidence was evaluated using the Grades of Recommendation, Assessment, Development, and Evaluation (GRADE) framework. **Results:** 18 systematic reviews met the inclusion criteria, encompassing 256 primary studies and 5092 participants. Overall methodological quality of the included reviews was rated as moderate to good. A variety of robotic and exoskeleton systems were noted across studies, with heterogeneous protocols and outcomes. Several reviews reported modest improvements in gait and postural balance; however, the findings were inconsistent, and pooled effects, where available, did not yield definitive conclusions regarding efficacy. **Conclusions:** Robotic and exoskeleton interventions may offer benefits for gait and postural balance in children and adolescents with CP, but the current evidence base remains inconclusive. Additional high-quality research is required to determine effectiveness more definitively.

## 1. Introduction

Cerebral palsy (CP) is defined as “a group of permanent disorders of the development of movement and posture, causing activity limitation, that are attributed to non-progressive disturbances that occurred in the developing fetal or infant brain [1]. The motor disorders of CP are often accompanied by disturbances of sensation, perception, cognition, communication, and behavior, by epilepsy, and by secondary musculoskeletal problems” [1]. The involvement of non-motor impairments such as intellectual disability, sensory deficits (e.g., visual and auditory impairments), and behavioral disorders (e.g., ADHD and autism spectrum disorders) renders CP a multidimensional condition. These comorbidities often exacerbate functional limitations and complicate care strategies. Although the underlying brain lesion is non-progressive, clinical symptoms may change over time due to growth, musculoskeletal adaptations, and environmental interactions [1].

CP represents the most prevalent cause of physical disability in childhood, with an incidence rate that has remained stable at approximately 2 to 3.5 cases per 1000 live births over the past four decades [2]. Individuals with CP frequently demonstrate impaired selective motor control and persistent synergistic movement patterns, which disrupt the development of coordinated gait mechanisms. These motor impairments are associated with compromised balance, reduced weight-bearing capacity during gait initiation, and deficits in limb stabilization [3], ultimately leading to gait instability, reduced endurance, and abnormal joint loading [4]. Motor dysfunction is a hallmark of CP, universally affecting all individuals with the diagnosis. Common motor impairments include muscle weakness, altered tone, contractures, and fatigue, which collectively disrupt gait kinematics by shortening step and stride lengths and reducing gait velocity [5]. Notably, approximately 90% of individuals with CP experience some form of ambulation difficulty [6,7]. Given the strong association between mobility and independence, improving pathological gait patterns remains a central therapeutic objective [7,8].

Although no definitive cure exists for CP, early and targeted interventions informed by the Gross Motor Function Classification System (GMFCS) are recommended to maximize neuroplasticity, minimize complications, and enhance function, participation, and quality of life for both children and caregivers [9]. The GMFCS is a widely adopted framework for categorizing the severity of gross motor impairment across five levels, based on age-specific and real-world performance [10,11]. Children classified within Levels I and II (approximately 62%) typically ambulate independently, while those in Level III (around 11%) often require assistive devices and may rely on wheelchairs for long distances [12]. Moreover, some individuals in GMFCS Level III may lose ambulatory capacity during adolescence or young adulthood [13,14,15,16,17,18], whereas those classified as Levels IV and V demonstrate severely limited or no ambulatory ability [13].

Rehabilitation strategies in CP emphasize goal-oriented, evidence-based approaches aimed at improving motor function [19,20]. High-evidence interventions include botulinum toxin A, constraint-induced movement therapy, bimanual training, and task-specific motor learning [19]. Additional modalities such as serial casting, orthopedic surgery, orthotic use, resistance training, stretching, hydrotherapy, and home-based programs are frequently employed to address specific motor deficits. Of these, repetitive, task-specific therapies have shown particular efficacy in improving motor outcomes [20,21].

In recent years, robotic-assisted rehabilitation has emerged as a promising and technologically advanced approach to improving gait and postural control in children with CP. This modality leverages principles of motor learning and neuroplasticity by delivering high-repetition, task-specific, and intensity-controlled therapeutic activities that may exceed what traditional therapy can provide [22]. One widely studied intervention is robot-assisted gait training (RAGT), which utilizes programmable robotic devices to facilitate consistent and repetitive lower limb movement patterns. These devices guide the child’s legs through pre-defined gait cycles, allowing for precise control of joint trajectories and walking cadence, thereby promoting neuromuscular re-education and facilitating the retraining of locomotor functions [22,23,24]. RAGT aims to optimize motor output by enhancing afferent sensory input and providing external feedback during task execution, which are critical elements in driving activity-dependent neuroplastic changes in the central nervous system [22]. The repetitive nature of robotic gait cycles reinforces correct motor patterns and enables motor learning, ultimately improving postural alignment, balance control, and walking efficiency. Similarly, robotic exoskeletons are designed to restore and support physiological gait kinematics by providing mechanical and/or electrical assistance to the hip, knee, and ankle joints. These devices not only enable active joint motion but also engage spinal and supraspinal locomotor circuits, modulating neural excitability and facilitating the reorganization of motor pathways involved in ambulation [4,8].

The implementation of robotic systems in pediatric neurorehabilitation offers several clinical advantages [25,26]. These include improved gait symmetry, enhanced endurance, and increased weight-bearing capacity, all of which are essential for achieving independent mobility and reducing secondary musculoskeletal complications in children with CP. Moreover, robotic-assisted training provides a standardized therapeutic dose, reduces therapist physical burden, and allows real-time monitoring of biomechanical parameters, thereby supporting individualized and data-driven rehabilitation planning [22]. Importantly, the structured and externally guided practice afforded by robotic devices complements conventional therapy by promoting functional independence and enhancing long-term mobility outcomes. Despite these potential benefits, the evidence regarding the efficacy of robotic and exoskeleton-based rehabilitation in pediatric CP populations remains inconclusive. Several systematic reviews have investigated these interventions, yet the findings are heterogeneous. While some reviews report statistically and clinically significant improvements in spatiotemporal gait parameters, postural balance, and gross motor function, others have found limited or negligible benefits compared to standard care or conventional therapy. These discrepancies may be attributable to variability in study design, sample characteristics, intervention protocols (e.g., device type, frequency, duration), and outcome measures across trials. Moreover, methodological limitations such as small sample sizes, lack of long-term follow-up, and inconsistent reporting of effect sizes further obscure the interpretability of the findings. Given these inconsistencies, there is a clear need to conduct a higher-level synthesis of the existing systematic reviews to determine the overall strength of evidence regarding robotic and exoskeletal rehabilitation in children and adolescents with CP. This study aims to systematically assess the available evidence from published systematic reviews concerning the effects and therapeutic benefits of robotic and exoskeleton interventions on gait and postural balance in children and adolescents with CP. By synthesizing high-quality evidence, this overview seeks to identify patterns in therapeutic outcomes and highlight potential methodological inconsistencies across reviews.

## 2. Materials and Methods

### 2.1. Study Design

This study was an overview of systematic reviews, and it was conducted in accordance with the recommendations published by Hunt et al. (2022) [8]. This study was registered (https://doi.org/10.17605/OSF.IO/DBY84) in Open Science Framework registries (OSF Registries) and followed Preferred Reporting Items for Systematic Reviews and Meta-Analyses (PRISMA) 2020 (Table 1) [27] to report our findings.

### 2.2. Literature Search

A comprehensive literature search was conducted in four databases: PubMed, CINAHL, Scopus, and The Cochrane Library to identify relevant studies up to May 2024. No restrictions on publication date were applied. The search was conducted in collaboration with a specialized librarian to ensure accuracy and comprehensiveness. The following keywords and Medical Subject Headings (MeSH) were used: (1) Gait [MeSH] OR Postural Balance [MeSH] OR Walk*; (2) Cerebral Palsy [MeSH] OR Hemiplegia [MeSH] OR Diplegia; (3) Exoskeleton Device [MeSH] OR Robotics [MeSH]; (4) Systematic Review [MeSH] OR Review. The searches in all databases were combined as follows: 1 AND 2 AND 3 AND 4.

### 2.3. Study Selection and Data Extraction

Two independent reviewers reviewed the titles of each article for initial selection. Abstracts of articles relevant to cerebral palsy, gait, and/or balance, and robotic/exoskeleton interventions were assessed, and full-text articles of eligible abstracts were retrieved for further examination. The same reviewers independently evaluated the full texts of the remaining articles and applied a predefined checklist to determine eligibility based on the following inclusion criteria: (i) a systematic review or meta-analysis that synthesized existing literature; (ii) studies including participants who were children or adolescents (0–18 years old) diagnosed with CP; (iii) reviews examining the use of robotic devices and/or exoskeletons for gait or postural balance rehabilitation; and (iv) articles published in English in peer-reviewed journals. Discrepancies between reviewers were resolved through consensus.

For data extraction, the same independent reviewers used a structured data extraction form to systematically collect relevant information. Extracted data included publication details (e.g., publication date, database search timeframe), study objectives, characteristics of included studies and populations, type of robotic devices used, primary findings related to gait and balance, and reported study limitations. Agreement between reviewers on study selection and data extraction was >93%.

### 2.4. Assessment of Methodological Quality of Included Studies

The methodological quality of the included reviews was assessed using the Assessment of Multiple Systematic Reviews (AMSTAR-2) appraisal tool (Table 2) [28]. AMSTAR-2, a 16-item tool widely utilized in systematic reviews, evaluates methodological rigor and has demonstrated acceptable inter-rater reliability, construct validity, and feasibility [29]. Two independent reviewers applied AMSTAR-2 following predefined criteria, and any discrepancies were resolved through discussion. In addition, the certainty of evidence across the included studies was assessed using the Grade of Recommendation, Assessment, Development, and Evaluation (GRADE) tool (Table 2). GRADE categorizes the strength of evidence on a four-point scale: high, moderate, low, and very low quality [30]. Final consensus among all authors was reached to resolve any remaining discrepancies in quality assessments. The agreement among the reviewers ranged from moderate to excellent (κ = 0.64–1.0).

## 3. Results

**Figure 1:**
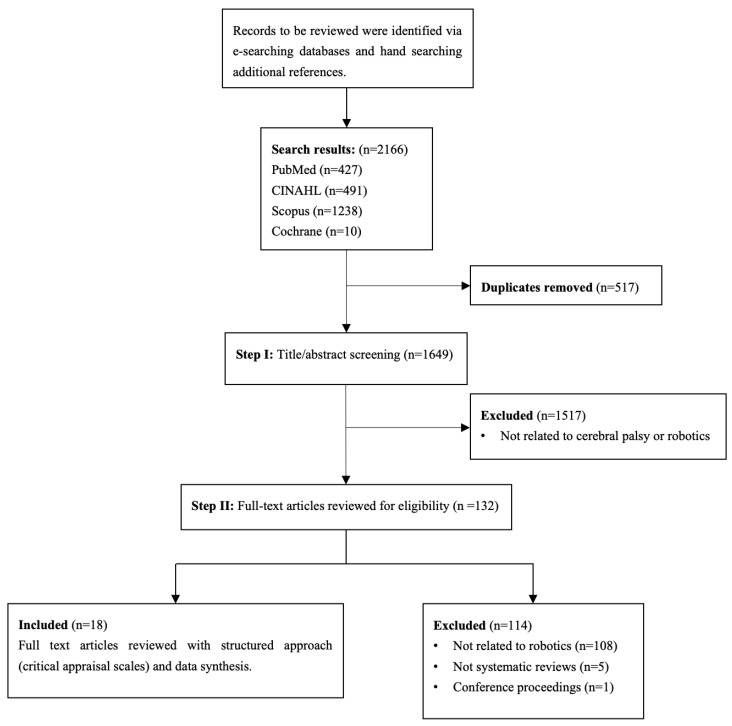
Flowchart of search and selection strategy.

The initial database search identified 2166 published studies related to systematic reviews examining the effects and therapeutic benefits of robotics and exoskeletons on gait and postural balance in children and adolescents with CP. After removing 517 duplicate records, 1649 studies remained for title and abstract screening (Figure 1). Following the initial screening process conducted by two independent reviewers, 1517 studies were excluded for not meeting the inclusion criteria, specifically for lacking relevance to CP or robotic-assisted rehabilitation. Subsequently, 132 full-text articles were retrieved and reviewed for final inclusion. Ultimately, 18 articles met the inclusion criteria (Figure 1), comprising four systematic reviews, five narrative reviews, and nine systematic reviews with meta-analyses [4,5,7,8,9,21,22,23,24,31,32,33,34,35,36,37,38,39].

### 3.1. Overview of Included Studies

The total number of participants across the included reviews exceeded 5092 individuals. However, one study did not report participant numbers [32], and another included pediatric populations beyond CP [37]. The included systematic reviews synthesized approximately 256 primary clinical studies; among these clinical studies, there were several studies that overlapped the different systematic reviews, with 145 unique studies. Most of the included reviews specified the study designs they assessed in their analyses: six reviews included randomized controlled trials (RCTs) [7,22,24,31,34,39], one review included case series and case studies [21], one review encompassed RCTs, cohort studies, case–control studies, and before-and-after studies [5], two reviews incorporated clinical controlled trials (CCTs) [9,35], two reviews combined RCTs and non-RCTs [23,36], one review included RCTs, non-RCTs, interrupted time-series designs, and pre/post-test designs without a control group [37], one review detailed RCTs, quasi-RCTs, and randomized cross-over trials [33], and one review examined RCTs, controlled and uncontrolled trials, and case series [38]. In contrast, three reviews did not specify the types of primary studies included in their analyses [4,8,32]. The publication date range of the studies within the included reviews spanned from 1980 to December 2022.

### 3.2. Quality of Systematic Reviews

AMSTAR-2 evaluates 16 domains, including protocol registration, duplicate processes, literature search comprehensiveness, and bias assessment methods. Each review was rated as critically low, low, moderate, or high based on overall compliance with AMSTAR-2 criteria.

Of the 18 included systematic reviews, five were rated as high quality: Alotaibi et al. (2024) [31], Cortés-Pérez et al. (2022) [35], Vézer et al. (2023) [39], Volpini et al. (2021) [23], and Wang et al. (2023) [24]. These reviews consistently met most AMSTAR-2 criteria, demonstrating rigorous methodology including comprehensive literature searches, duplicate data extraction, appropriate risk of bias assessment, and transparent reporting. Five reviews were rated as moderate quality, including Chiu et al. (2020) [33], Cumplido et al. (2021) [36], Hunt et al. (2022) [8], Lefmann et al. (2017) [37], and Qian et al. (2023) [22]. These studies generally demonstrated sound methodology but had some limitations, such as incomplete reporting of excluded studies or a lack of protocol registration. Four reviews were rated as low quality: Carvalho et al. (2017) [5], Llamas-Ramos et al. (2022) [7], Olmos-Gómez et al. (2021) [9], and Valè et al. (2021) [38]. These studies met several core methodological standards but lacked robustness in key areas such as bias handling and synthesis transparency. The remaining four studies: Bonanno et al. (2023) [32], Bunge et al. (2021) [21], Conner et al. (2022) [34], and Vova et al. (2019) [4] were rated as critically low quality due to multiple methodological shortcomings. Common issues in these studies included unclear eligibility criteria, absence of protocol registration, inadequate risk of bias assessment, and insufficient reporting of funding sources or excluded studies.

The quality of evidence reported across the included systematic reviews was appraised using the GRADE framework, which considers factors such as study design, risk of bias, inconsistency, indirectness, imprecision, and publication bias. Each review was assigned an evidence level of high, moderate, or low. Most studies (n = 10) were rated as providing low-quality evidence [4,5,8,9,22,32,34,35,36,38]. These reviews commonly exhibited limitations such as small sample sizes, high heterogeneity in interventions and populations, and methodological concerns related to study design or incomplete reporting. Six studies were assessed as offering moderate-quality evidence [7,21,24,31,37,39], typically due to moderate study limitations or variability in outcomes that slightly reduced confidence in the estimates. Only two reviews were graded as providing high-quality evidence [23,33], characterized by consistent findings, low risk of bias, and robust methodologies, lending strong support to their conclusions. This distribution of GRADE scores highlights the current limitations in the evidence base regarding robotic and exoskeleton interventions in CP rehabilitation, underscoring the need for more rigorously designed trials with standardized protocols and longer-term follow-up (Table 2).

### 3.3. Effectiveness of Robot-Assisted Gait Training (RAGT)

Evidence suggests that RAGT is effective in improving motor function in children and adolescents with CP [9]. Several studies reported significant improvements following RAGT, particularly in temporospatial gait parameters, gait kinematics, and functional mobility tests, including the Six-Minute Walk Test (6 MWT) and 10-Meter Walk Test (10 MWT) [5,23,24,32]. Improvements were also observed in the standing and walking dimensions (D and E) of the GMFM [5,22,23,24,32]. Studies further suggest that individuals with GMFCS levels I or II experience greater motor function improvements than those classified under GMFCS levels III or IV [5].

### 3.4. Impact of Robotic Exoskeletons on Mobility in CP

Robotic exoskeletons have demonstrated measurable effects on gait and postural balance outcomes in children with CP. Several reviews have identified improvements in spatiotemporal parameters, such as gait velocity, cadence, step length, and stride length, following exoskeleton-assisted training [8,21,32]. Interventions using Lokomat and Alter-G were most frequently reported and were linked to increased walking distance, improved gait symmetry, and enhanced endurance [32]. Studies that employed powered lower-limb exoskeletons (PoLLE) also noted reductions in energy expenditure and metabolic cost, as measured by the Physiological Cost Index and Borg scale [21]. Other reviews reported benefits in postural balance and stability, including improvements on the Berg Balance Scale (BBS) and Timed Up and Go (TUG) test [8]. Exoskeletons designed to facilitate knee and hip extension during stance were associated with improved dynamic balance and reduced gait-related metabolic demand [32]. Lightweight or untethered exoskeletons further demonstrated favorable changes in spatiotemporal gait parameters, suggesting enhanced gait efficiency during training [8].

### 3.5. Comparing RAGT and Traditional Therapy

The included reviews reported mixed outcomes when comparing RAGT and exoskeleton interventions with traditional or treadmill-based physiotherapy. Powered body-weight–supported treadmill training (PBWSTT) was reported to increase gait speed, gross motor skills, stability, balance, and walking ability compared with resistance training over 4–12 weeks [31]. Additionally, PBWSTT was ranked as the highest for improving gait velocity, while RAGT ranked highest for GMFM-88 Dimension D (standing) and Dimension E (walking, running, and jumping) [22]. Another review [39] reported changes in step length, gait velocity, and postural balance after RAGT that were not statistically different from those seen with treadmill or balance training. Similarly, one review [23] identified increases in walking distance and gait speed after RAGT, with no statistically significant differences from conventional rehabilitation. Small improvements were observed in walking speed and gross motor function following mechanically assisted walking training without body-weight support compared with no training, but little to no difference when compared with overground walking or training with body-weight support [33]. Two reviews [9,34] found no significant differences in walking speed, walking distance, or gross motor function between RAGT, RAGT combined with physiotherapy, and dose-matched conventional physiotherapy. Additionally, significant improvements were reported in GMFM-88 D and E scores, Berg Balance Scale, and 6-Minute Walk Test results for RAGT relative to control groups, while differences in gait speed were not statistically significant [24]. Gait speed, endurance, muscle strength, and balance were reported to increase following robotic or functional electrical stimulation interventions compared with standard physiotherapy [4,7].

## 4. Discussion

This review synthesized evidence from 18 systematic reviews examining the therapeutic effects of RAGT and exoskeleton therapy on gait and postural balance in children and adolescents with CP (Table 3). RAGT was linked to moderate yet clinically significant enhancements in gross motor function, especially in GMFM dimensions D and E, as well as gait speed, endurance, and walking distance. The methodological quality of the included studies was variable, with significant variation in research design, training regimens, outcome measures, and participant characteristics, which constrained the generalizability of the findings. The devices most extensively examined were Lokomat, Robogait, and CP-Walker, which exhibited considerable differences in control tactics and degrees of user engagement.

### 4.1. Functional and Design Considerations of Robotic and Exoskeleton Systems

RAGT and exoskeleton systems, particularly when paired with BWS and treadmill-based protocols, offer a high-intensity, task-specific, and repetitive training environment (factors known to facilitate motor learning and promote neuroplasticity in children with CP) [40]. RAGT induces significant changes in cortical activation patterns, particularly in the prefrontal and sensorimotor regions, supporting the notion that these interventions promote functional neuroplasticity in pediatric populations [40]. Additionally, the use of BWS systems varies in appropriateness depending on motor function severity. For children with GMFCS levels I–II, BWS may be unnecessary and could even hinder gait learning by reducing motor demands. In contrast, children at GMFCS levels III–IV may benefit significantly from BWS, as it aids verticalization, trunk stabilization, and reduces fall risk during gait training [41,42]. Despite these promising findings, the degree of volitional control permitted by robotic systems critically influences their therapeutic impact. Passive or fully assistive modes have been shown to limit engagement of motor learning circuits. Conversely, resistive or actively guided training modalities (those that demand user initiation or response) appear more effective in promoting neurorehabilitation [34]. Furthermore, classification between assistive and rehabilitative robotic devices remains ambiguous in the literature. Some systems, such as the Ekso-GT, were primarily designed to substitute motor function and are less interactive, while others explicitly aim to foster neuroplasticity through repetitive, task-oriented practice [7]. This reflects broader evidence emphasizing the necessity of volitional effort and cortical activation for functional recovery in CP [8,33]. It is crucial to understand that Robotic systems differ in their mechanical architecture (e.g., end-effector vs. wearable exoskeleton), degrees of freedom, and feedback mechanisms (e.g., visual, haptic, auditory), which in turn influence usability and outcomes [43]. Wearable exoskeletons, in particular, align with the user’s joint anatomy and can be designed to deliver assistive, resistive, or active-guided support across the hip, knee, and ankle [44]. However, one of the primary challenges in pediatric applications is the lack of devices tailored to children’s anthropometrics. Many systems were originally designed for adults and lack adjustability in size, weight, and force output [38,45]. This limitation restricts their use in younger or smaller children, emphasizing the need for pediatric-specific design. Device mass is also a critical consideration. Studies suggest that masses exceeding 2.5 kg may negatively affect gait kinematics and increase the metabolic cost of walking in children with CP [46,47]. Notably, several pediatric exoskeletons such as the tethered knee exoskeleton and P.REX have demonstrated beneficial kinematic effects, including improved knee and hip extension during the stance phase, especially in children with crouch gait [48,49,50,51,52,53]. Nevertheless, bulky systems or those with passive ankle joints may compromise natural gait mechanics or increase fatigue [47,54].

### 4.2. Methodological and Technical Gaps

The current literature is limited by inconsistencies in training parameters, including frequency, intensity, session length, and overall intervention duration. Reviews highlight that intervention periods range widely (2–12 weeks), with treatment frequencies from 2 to 5 days per week, which likely contributes to variable outcomes [23]. Moreover, several studies lacked stratification by GMFCS level, which may obscure differential responses across severity groups. Indeed, emerging evidence suggests that children with greater motor impairment (GMFCS III–IV) derive more pronounced benefits from RAGT, particularly in balance, aerobic fitness, and endurance [23,36]. Technologically, most current systems are tethered, with limited adaptability to user-generated inputs. Few studies employed devices with real-time adaptive controllers, which are crucial for promoting user-driven neuroplastic change [8]. Additionally, the use of virtual reality and biofeedback (though promising in improving engagement and motivation) remains underreported and inconsistently applied [37,55]. The lack of consistent reporting on device characteristics, setup, and progression algorithms further impairs synthesis efforts.

### 4.3. Limitations and Uncertainties in RAGT and Exoskeleton Research

There remains limited evidence regarding the efficacy of weight-unsupported arm and knee mechanisms (WUAM and WUAKM) for improving gait in children with CP [8]. Network meta-analyses indicate that PBWSTT may be the most effective intervention for increasing gait velocity, whereas RAGT appears to yield the greatest improvements in GMFM scores. However, PBWSTT remains the intervention with the highest likelihood of effectiveness for gait improvement [22]. Meta-analyses by Wang et al. (2023) [24] have demonstrated that RAGT significantly outperforms conventional rehabilitation in improving motor function in individuals with CP. Specifically, RAGT has been associated with greater improvements in the GMFM-8 D and E area scores, BBS and 6MWT performance [24]. However, another meta-analysis by Volpini M et al. (2021) [23] found no significant differences between RAGT alone, RAGT combined with physical therapy, and physical therapy alone in terms of overall gait characteristic improvements [9]. Additionally, the magnitude of effect size estimates was consistently low or very low across all comparisons examined, suggesting that while RAGT shows some benefits, its superiority over conventional rehabilitation approaches remains inconclusive [9]. Furthermore, due to insufficient data, no definitive conclusions could be drawn regarding the impact of RAGT on gait stability, specifically changes in step width and step length, when compared to control groups. As a result, the effectiveness of RAGT in improving gait stability remains uncertain.

The most comprehensive and up-to-date meta-analysis of RAGT rehabilitation techniques suggests that, despite being designed to harness neuroplasticity through forced repetitive gait movements, RAGT does not consistently outperform traditional physiotherapy or treadmill-based training in improving gait function in children with CP. The therapeutic benefits of RAGT, as measured by functional and biomechanical outcomes, were comparable to those achieved with physiotherapy combined with treadmill training [34]. Other studies have similarly concluded that robotic-assisted training does not provide superior outcomes compared to equivalent amounts of treadmill training or balance training [39]. While RAGT has shown promise in improving specific motor function parameters, its overall effectiveness remains uncertain, with limited evidence supporting its ability to significantly enhance gait speed and reduce muscle spasticity [7,24,36].

### 4.4. Clinical Implications

RAGT and exoskeleton therapy should be understood as complementary to, rather than replacements for, conventional rehabilitation therapy. Across the included reviewed evidence, these technologies show their greatest value when integrated into individualized, multidisciplinary programs that emphasize repetition, task-specific, and active motor engagement. Robotic systems can help standardize high-intensity training, reduce therapist workload, and maintain patient motivation, especially when paired with virtual reality or biofeedback components that make therapy more engaging and responsive [5,8,9,23,33,36,38].

#### 4.4.1. Children with Mild to Moderate Impairment (GMFCS II–III)

For ambulatory children in GMFCS levels II–III, RAGT and PBWSTT appear most effective when delivered at moderate intensity and frequency sufficient to stimulate motor learning. and several reviews [5,22,23,24,31,39] suggested that programs involving approximately 30 to 45 min per session, 3 to 5 times per week, over 8 to 12 weeks lead to measurable improvements in gait speed, endurance, and postural control. Gradually reducing robotic assistance while encouraging active participation and using resistive or “assist-as-needed” modes enhances cortical activation and functional transfer [8,33,34]. Progress should be tracked with standardized outcome measures such as GMFM Dimentions D and E, the 6-Minute Walk Test, and the Berg Balance Scale [5,23,24].

#### 4.4.2. Children with Severe Motor Impairment (GMFCS IV–V)

Although children classified within GMFCS levels IV–V may show limited gains in independent ambulation, RAGT and exoskeletal devices still provide substantial physiological and preventive benefits. The reported advantages included safer verticalization, improved cardiopulmonary endurance, maintenance of joint range, and lower risk of secondary musculoskeletal complications such as contractures [7,8,36,38]. Studies involving this subgroup typically use between 20 and 45 min per session, 2 to 5 times per week, for 4 to 12 weeks, with progressive adjustment of body-weight support and walking speed to match tolerance [21,36,37]. However, the literature does not yet establish an optimal dose–response relationship for this population. Accordingly, session duration and intensity should be tailored to each child, emphasizing comfort, hemodynamic stability, and safety rather than strict performance targets. For non-ambulatory children, low-intensity, progressively adaptive programs that focus on postural control and assisted stepping may still offer important circulatory and psychosocial benefits.

In practice, robotic and exoskeleton training should be embedded within comprehensive rehabilitation plans that also address strengthening, balance, and functional mobility. Their principal contribution lies in intensifying task-specific practice and reinforcing neuroplasticity through repetitive, feedback-guided movement. Because evidence on long-term outcomes and cost-effectiveness remains limited, ongoing monitoring, individualized program design, and follow-up are essential to ensure that these technologies deliver meaningful and sustainable benefits in real-world clinical settings.

### 4.5. Recommendations for Future Research

High-quality RCTs are needed to establish definitive efficacy, particularly trials that stratify participants by GMFCS level and age group. Future studies should also evaluate the impact of adaptive control strategies, untethered systems, and VR or gamified feedback on both functional and cortical outcomes [33,36]. Standardization in outcome reporting, using biomechanical (e.g., joint angles, spatiotemporal metrics), neurophysiological (e.g., EMG), and psychosocial measures, is urgently needed to enhance cross-study comparability [4,8]. Furthermore, trials should include cost-effectiveness evaluations, real-world feasibility analyses, and reporting on adverse events and acceptability to families. There is also a growing need to examine developmental timing effects (i.e., whether early intervention during peak neuroplastic windows yields superior outcomes compared to later stages of development) [37]. Finally, creating a core outcome set for robotic gait rehabilitation in pediatric CP populations would significantly strengthen evidence synthesis and guide clinical decision-making.

## 5. Study Limitations

This study has several limitations that should be acknowledged. First, the literature search was restricted to English-language publications, which may have led to the exclusion of relevant studies published in other languages, potentially limiting the comprehensiveness of the review. Additionally, the generalizability of the findings is constrained, as many included studies focused on specific subpopulations of individuals with CP, making it difficult to apply the results broadly across diverse patient groups. Another key limitation is the variability in the robotic devices evaluated, as some studies assessed specific robotic systems without providing comparative analyses across different technologies. This lack of direct comparison hinders the ability to determine which robotic interventions are most effective. Furthermore, the heterogeneity in robotic and exoskeleton interventions presents challenges in drawing generalized recommendations, as the reviewed studies incorporated a wide range of devices with differing mechanisms, training protocols, and therapeutic goals. Addressing these limitations requires high-quality, standardized clinical trials with larger sample sizes, extended follow-up periods, and cost-effectiveness assessments to better understand the long-term impact of robotic-assisted training in CP rehabilitation.

## 6. Conclusions

This systematic review aimed to assess the effectiveness of robotic and exoskeleton training for gait and balance rehabilitation in individuals with CP. While many studies reported modest improvements in gait parameters (e.g., gait speed, step length, walking distance) and gross motor function (GMFM-D and GMFM-E), the overall evidence remains inconclusive. Most included studies were of low or moderate quality, limiting the strength of conclusions regarding RAGT and exoskeleton therapy. The effectiveness of robotic-assisted therapy compared to traditional rehabilitation remains uncertain, as some studies indicate positive outcomes, while others find no significant differences. Robotic training should not be considered a standalone intervention but rather an adjunct to conventional therapy. Future research should prioritize high-quality RCTs with larger sample sizes and longer follow-ups to provide more definitive evidence on the efficacy of robotic and exoskeleton training in CP rehabilitation. Additionally, optimizing training protocols (e.g., intensity, duration, and frequency) is essential to maximize clinical benefits. In conclusion, robotic and exoskeleton training show promise as therapeutic tools for CP rehabilitation; however, further research is needed to fully determine their benefits, limitations, and long-term effectiveness.

## Figures and Tables

**Table 1:** PRISMA 2020 Checklist.

Section and Topic	Item #	Checklist Item	Location Where Item Is Reported
**Title**			
Title	1	Identify the report as a systematic review.	Title
**Abstract**			
Abstract	2	See the PRISMA 2020 for Abstracts checklist.	Abstract
**Introduction**			
Rationale	3	Describe the rationale for the review in the context of existing knowledge.	Lines 101–121
Objectives	4	Provide an explicit statement of the objective(s) or question(s) the review addresses.	Lines 122–128
**Methods**			
Eligibility criteria	5	Specify the inclusion and exclusion criteria for the review and how studies were grouped for the syntheses.	Lines 149–154
Information sources	6	Specify all databases, registers, websites, organisations, reference lists and other sources searched or consulted to identify studies. Specify the date when each source was last searched or consulted.	Lines 134–142
Search strategy	7	Present the full search strategies for all databases, registers and websites, including any filters and limits used.	Lines 134–142
Selection process	8	Specify the methods used to decide whether a study met the inclusion criteria of the review, including how many reviewers screened each record and each report retrieved, whether they worked independently, and if applicable, details of automation tools used in the process.	Lines 144–153
Data collection process	9	Specify the methods used to collect data from reports, including how many reviewers collected data from each report, whether they worked independently, any processes for obtaining or confirming data from study investigators, and if applicable, details of automation tools used in the process.	Lines 155–159
Data items	10a	List and define all outcomes for which data were sought. Specify whether all results that were compatible with each outcome domain in each study were sought (e.g., for all measures, time points, analyses), and if not, the methods used to decide which results to collect.	Lines 155–159
	10b	List and define all other variables for which data were sought (e.g., participant and intervention characteristics, funding sources). Describe any assumptions made about any missing or unclear information.	Lines 155–159
Study risk of bias assessment	11	Specify the methods used to assess risk of bias in the included studies, including details of the tool(s) used, how many reviewers assessed each study and whether they worked independently, and if applicable, details of automation tools used in the process.	Lines 161–171
Effect measures	12	Specify for each outcome the effect measure(s) (e.g., risk ratio, mean difference) used in the synthesis or presentation of results.	N/A
Synthesis methods	13a	Describe the processes used to decide which studies were eligible for each synthesis (e.g., tabulating the study intervention characteristics and comparing against the planned groups for each synthesis (item #5)).	N/A
	13b	Describe any methods required to prepare the data for presentation or synthesis, such as handling of missing summary statistics, or data conversions.	N/A
	13c	Describe any methods used to tabulate or visually display results of individual studies and syntheses.	Table 1 and Table 2
	13d	Describe any methods used to synthesize results and provide a rationale for the choice(s). If meta-analysis was performed, describe the model(s), method(s) to identify the presence and extent of statistical heterogeneity, and software package(s) used.	Table 1 and Table 2
	13e	Describe any methods used to explore possible causes of heterogeneity among study results (e.g., subgroup analysis, meta-regression).	N/A
	13f	Describe any sensitivity analyses conducted to assess robustness of the synthesized results.	N/A
Reporting bias assessment	14	Describe any methods used to assess risk of bias due to missing results in a synthesis (arising from reporting biases).	Table 2
Certainty assessment	15	Describe any methods used to assess certainty (or confidence) in the body of evidence for an outcome.	N/A
**Results**			
Study selection	16a	Describe the results of the search and selection process, from the number of records identified in the search to the number of studies included in the review, ideally using a flow diagram.	Results: Lines 1–12
	16b	Cite studies that might appear to meet the inclusion criteria, but which were excluded, and explain why they were excluded.	N/A
Study characteristics	17	Cite each included study and present its characteristics.	Table 1 and Table 2
Risk of bias in studies	18	Present assessments of risk of bias for each included study.	Table 2
Results of individual studies	19	For all outcomes, present, for each study: (a) summary statistics for each group (where appropriate) and (b) an effect estimate and its precision (e.g., confidence/credible interval), ideally using structured tables or plots.	N/A
Results of syntheses	20a	For each synthesis, briefly summarise the characteristics and risk of bias among contributing studies.	Table 2
	20b	Present results of all statistical syntheses conducted. If meta-analysis was done, present for each the summary estimate and its precision (e.g., confidence/credible interval) and measures of statistical heterogeneity. If comparing groups, describe the direction of the effect.	N/A
	20c	Present results of all investigations of possible causes of heterogeneity among study results.	Table 2
	20d	Present results of all sensitivity analyses conducted to assess the robustness of the synthesized results.	N/A
Reporting biases	21	Present assessments of risk of bias due to missing results (arising from reporting biases) for each synthesis assessed.	N/A
Certainty of evidence	22	Present assessments of certainty (or confidence) in the body of evidence for each outcome assessed.	N/A
**Discussion**			
Discussion	23a	Provide a general interpretation of the results in the context of other evidence.	Lines 166–174
	23b	Discuss any limitations of the evidence included in the review.	Lines 256–271
	23c	Discuss any limitations of the review processes used.	Lines 256–271
	23d	Discuss implications of the results for practice, policy, and future research.	Lines 273–288
**Other Information**			
Registration and protocol	24a	Provide registration information for the review, including register name and registration number, or state that the review was not registered.	Lines 131–134
	24b	Indicate where the review protocol can be accessed, or state that a protocol was not prepared.	Lines 131–134
	24c	Describe and explain any amendments to information provided at registration or in the protocol.	N/A
Support	25	Describe sources of financial or non-financial support for the review, and the role of the funders or sponsors in the review.	Line 296
Competing interests	26	Declare any competing interests of review authors.	Line 297
Availability of data, code and other materials	27	Report which of the following are publicly available and where they can be found: template data collection forms; data extracted from included studies; data used for all analyses; analytic code; any other materials used in the review.	N/A

**Table 2:** Quality assessment (AMSTAR) of included systematic reviews.

Author and Year	AMSTAR Criteria																
	1	2	3	4	5	6	7	8	9	10	11	12	13	14	15	16	Total Score
Alotaibi et al., 2024 [31]	Y	Y	Y	PY	Y	Y	PY	Y	Y	N	Y	Y	Y	Y	Y	Y	High
Bonanno et al., 2023 [32]	Y	PY	N	PY	Y	N	PY	N	N	N	NMA	NMA	N	N	NMA	Y	Critically Low
Bunge et al., 2021 [21]	Y	PY	N	PY	Y	Y	PY	PY	N	N	NMA	NMA	N	Y	NMA	Y	Critically Low
Carvalho et al., 2017 [5]	Y	Y	N	PY	Y	Y	PY	PY	N	N	Y	N	N	Y	N	Y	Low
Chiu et al., 2020 [33]	Y	Y	Y	PY	Y	Y	Y	PY	Y	Y	NMA	NMA	Y	Y	NMA	Y	Moderate
Conner et al., 2022 [34]	Y	Y	Y	PY	Y	Y	PY	PY	N	N	Y	N	N	Y	N	Y	Critically Low
Cortés-Pérez et al., 2022 [35]	Y	Y	Y	PY	Y	Y	PY	Y	Y	Y	N	Y	Y	Y	Y	Y	High
Cumplido et al., 2021 [36]	Y	PY	Y	PY	Y	Y	PY	PY	Y	N	NMA	NMA	Y	Y	NMA	Y	Moderate
Hunt et al., 2022 [8]	Y	PY	Y	PY	Y	Y	PY	PY	Y	N	NMA	NMA	Y	N	NMA	Y	Moderate
Lefmann et al., 2017 [37]	Y	PY	Y	PY	Y	Y	PY	PY	Y	N	NMA	NMA	Y	N	NMA	Y	Moderate
Llamas-Ramos et al., 2022 [7]	Y	PY	Y	PY	Y	Y	PY	PY	Y	N	NMA	NMA	N	N	NMA	Y	Low
Olmos-Gómez et al., 2021 [9]	Y	PY	Y	PY	Y	Y	PY	PY	Y	N	Y	Y	Y	N	N	Y	Low
Qian et al., 2023 [22]	Y	PY	Y	Y	Y	Y	PY	PY	Y	N	Y	Y	Y	Y	Y	Y	Moderate
Valè et al., 2021 [38]	Y	PY	Y	PY	Y	Y	PY	PY	Y	N	NMA	NMA	Y	N	NMA	Y	Low
Vezér et al., 2024 [39]	Y	Y	Y	PY	Y	Y	PY	PY	PY	N	Y	Y	Y	Y	Y	Y	High
Volpini et al., 2022 [23]	Y	Y	Y	PY	Y	Y	PY	PY	Y	N	Y	Y	Y	Y	Y	Y	High
Vova et al., 2019 [4]	Y	N	N	PY	N	N	PY	PY	N	N	NMA	NMA	N	N	NMA	Y	Critically Low
Wang et al., 2023 [24]	Y	Y	Y	PY	Y	Y	PY	Y	Y	N	Y	Y	Y	Y	Y	Y	High

AMSTAR: A Measurement Tool to Assess Systematic Reviews; Y: Yes (met the criteria); PY: Partial Yes (met the criteria partially); N: No (did not meet the criteria). NMA: No meta-analysis conducted. Item 1: Did the research questions and inclusion criteria for the review include the components of PICO?; Item 2: Did the report of the review contain an explicit statement that the review methods were established prior to the conduct of the review, and did the report justify any significant deviations from the protocol?; Item 3: Did the review authors explain their selection of the study designs for inclusion in the review?; Item 4: Did the review authors use a comprehensive literature search strategy?; Item 5: Did the review authors perform study selection in duplicate?; Item 6: Did the review authors perform data extraction in duplicate?; Item 7: Did the review authors provide a list of excluded studies and justify the exclusions?; Item 8: Did the review authors describe the included studies in adequate detail?; Item 9: Did the review authors use a satisfactory technique for assessing the risk of bias (RoB) in individual studies that were included in the review?; Item 10: Did the review authors report on the sources of funding for the studies included in the review?; Item 11: If meta-analysis was performed, did the review authors use appropriate methods for statistical combination of results?; Item 12: If meta-analysis was performed, did the review authors assess the potential impact of RoB in individual studies on the results of the meta-analysis or other evidence synthesis?; Item 13: Did the review authors account for RoB in individual studies when interpreting/discussing the results of the review?; Item 14: Did the review authors provide a satisfactory explanation for, and discussion of, any heterogeneity observed in the results of the review?; Item 15: If they performed quantitative synthesis, did the review authors carry out an adequate investigation of publication bias (small study bias) and discuss its likely impact on the results of the review?; Item 16: Did the review authors report any potential sources of conflict of interest, including any funding they received for conducting the review?

**Table 3:** Summary of Studies.

Authors	Review Objective	Participants	No. of Included Studies	Robotics Included in the Review	Main Findings	GRADE
Alotaibi et al., 2024 [31]	To review the scientific literature on the efficacy of PBWSTT on various outcome measures among children with CP in different settings.	255	10	PBWSTT	PBWSTT programs of 4–12 weeks produced consistent improvements in gait, gross motor function, stability and balance in children and adolescents with CP. PBWSTT proved feasible across settings (home, school). Evidence is sparse for longer programs (12–24 weeks) and long-term retention.	Moderate
Bonanno et al., 2023 [32]	To investigate the effects of robotic systems on improving gait and balance in children and adolescents with CP, with a focus on biomechanical implications.	Not specified	18	Lokomat, Alter-G, HBRS, RG, HWA, RAGT, InnPro and Exoskeleton	The review reports that robotic-assisted gait and trunk training (RAGT) and related devices (exoskeletons, Alter-G, HBRS) can improve postural control, balance, trunk activation, and gross motor function in children and adolescents with CP—especially those with mild–moderate impairments. Robotic approaches tend to better improve dynamic balance and gait symmetry than some spatiotemporal parameters. Combining robotics with VR may boost outcomes. However, heterogeneous devices, variable protocols, and limited participant detail make it hard to identify the single best approach.	Low
Bunge et al., 2021 [21]	To determine the effectiveness of utilizing powered lower limb exoskeletons on gait in individuals with CP.	82	13	HAL, CPW, LEE, WAKE, BLAE, and NFW	The evidence indicates beneficial changes in spatiotemporal gait outcomes after interventions, with 10 of 13 studies reporting higher gait velocity (5 reaching statistical significance). Findings on metabolic/energy cost are inconsistent—some studies report reductions while others find no change. Reported adverse events (skin irritation, fatigue) were few and transient. Major limitations are small pilot samples and considerable participant heterogeneity (notably across GMFCS levels), which reduce generalizability.	Moderate
Carvalho et al., 2017 [5]	To review the effects of robotic gait training practices in individuals with cerebral palsy.	189	10	GTI and Locomat	Review shows that robotic gait training modestly improves gait speed and endurance and yields small improvements in gross motor function. Higher training dose (≥4 sessions/week and ≥30 min/session) is associated with larger gains. However, small sample sizes, broad GMFCS ranges (I–IV), and inconsistent training parameters across studies weaken causal conclusions.	Low
Chiu et al., 2020 [33]	To assess the effects of mechanically assisted walking training compared to control for walking, participation, and quality of life in children with cerebral palsy 3 to 18 years of age.	451	17	RAGT	The reviews report limited-to-modest benefits from mechanically assisted walking interventions in children with CP. Body-weight-supported robotic training increased walking speed compared with no training but generally did not improve gross motor function or outperform overground walking. Study heterogeneity and sparse representation of GMFCS IV and children < 9 years limit confidence.	High
Conner et al., 2022 [34]	To determine if robotic gait training for individuals with CP is effective more than standard physical therapy care.	188	8	Lokomat, GTI and 3DCaLT	The review found limited additional benefit of robotic gait devices versus dose-matched conventional therapy for walking speed and gross motor function, likely because many devices are primarily assistive and do not sufficiently engage active neuromuscular control. Resistive robotic training produced better locomotor gains. VR and biofeedback appear to enhance outcomes when paired with robotics. Interpretation is constrained by small samples, diverse protocols, high CP heterogeneity, and the review’s focus on spastic diplegia/triplegia.	Low
Cortés-Pérez et al., 2022 [35]	To review previous studies related to RAGT and compare its efficacy against CT or TT for improving gait ability, gross motor function and functional independence in children with CP.	413	15	Lokomat, walkbot-k, EksoGT, RT600, GTI, 3DCaLT, InnPro	The review indicates RAGT yields superior post-intervention gains in gait speed, walking distance and GMFM walking/running/jumping compared with dose-matched conventional therapy. Comparisons with treadmill training are generally inconclusive, and no consistent benefits were seen for step length or when RAGT was added to conventional therapy. Confidence is tempered by small study counts, risk of publication bias, and limited long-term follow-up.	Low
Cumplido et al., 2021 [36]	To assess the safety and efficacy of robot-assisted interventions in rehabilitation programs for children with CP or SMA.	742	21	Locomat, CP walker, Walkbot-K, The Hybrid Assistive Limb, Robogait	The review reports that robotic-assisted gait training is generally well tolerated in pediatric CP populations and that gait exoskeletons (e.g., Lokomat) show promising gait improvements in some studies. However, results vary by protocol and participant characteristics, and confidence is limited by small sample sizes, short or absent follow-up, unclear randomization and control procedures, and narrow selection criteria that may have excluded relevant evidence.	Low
Hunt et al., 2022 [8]	To systematically review the literature investigating spatiotemporal, kinematic, kinetic, muscle activity and physiological parameters during exoskeleton-assisted walking in children with CP.	25	13	TKE, TAE, UTAE, WUAM, WUKAM, P.REX, UAE	The literature suggests lightweight exoskeletons can improve stance-phase knee/hip extension, enhance step length and other spatiotemporal gait measures, and sometimes lower walking energy cost in children with CP. TKE has repeatedly increased walking speed (primarily via longer stride length). UAE, TKE and P.REX show promise for crouch gait. Overall evidence is sparse, based on small studies with varied protocols, which makes direct comparison and generalization difficult.	Low
Lefmann et al., 2017 [37]	To identify and appraise the existing evidence for the effectiveness of RAGT for pediatric gait disorders, including modes of delivery and potential benefit.	468	17	Lokomat, GTI and CPW	The review finds no consistent benefit of RAGT versus conventional physiotherapy for gait function at group level (meta-analysis showed no significant difference). A minority of studies reported durable or endurance improvements (3 of 8 for endurance), but results are variable. Combining RAGT with VR often produced larger effects, suggesting increased engagement matters. Overall confidence is low because of small sample sizes, low methodological quality, high risk of bias, and heterogeneous RAGT protocols.	Moderate
Llamas-Ramos et al., 2022 [7]	To systematically review the available evidence on the effectiveness of robotic systems for the treatment of children with CP to improve their autonomy and quality of life.	174	7	Lokomat, InnPro, robogait, WKS	Review concluded that across studies, adding robotic devices to conventional therapy produced small improvements in gait, strength, balance and endurance, with some sustained effects reported for Innowalk Pro. The Lokomat (which was the most studied) brought only modest gains and did not clearly beat standard therapy. Importantly, robotics serve best as complements to usual care. Evidence is constrained by short follow-ups, low methodological quality, and small sample sizes, so larger, longer, rigorous trials are needed.	Moderate
Olmos-Gómez et al., 2021 [9]	To examine the efficacy of RAGT among children and adolescents with CP to improve both standing and walking, as well as the characteristics of the gait with respect to speed, endurance, and stride length.	217	8	Lokomat, 3DCaLT, InnPro, Gait trainer.	Although some individual studies hint that RAGT can help children and adolescents with CP, the pooled analyses show no clear superiority of RAGT (alone or combined with physiotherapy) compared with physiotherapy alone across examined gait outcomes. Reported effects are small. Major limits are small study counts, strict selection criteria causing clinical heterogeneity, variable devices/doses/protocols, and unclear/high risk of bias, all of which restrict clinical recommendations.	Low
Qian et al., 2023 [22]	To evaluate and compare the effects of different approaches of gait training on gait ability in people with CP.	516	20	PBWSTT and RAGT	The meta-analysis suggests BWSTT is most likely to improve gait speed, whereas robotic-assisted approaches (RAGT) are most likely to improve standing (GMFM-D) and walking/running/jumping (GMFM-E). Effect-ranking (SUCRA) favors different interventions for different outcomes, but sparse study counts and inconsistent reporting of total training duration, session frequency and exercise intensity weaken the certainty and practical applicability of these findings.	Low
Valè et al., 2021 [38]	To provide an overview of the existing literature on robots and electromechanical devices used for the rehabilitation of gait and balance among children with neurological disabilities.	29	31	Lokomat, Lokomat +FreeD, MOTOMED, HAL, Ekso GT, IRAP, InnPro, Trexo-Home, GTI, FORTIS-102, PediAnklebot, ATLAS Exoskeleton	The review finds robot-assisted therapy feasible and potentially beneficial for children and adolescents, with few reported adverse events. It highlights the need for pediatric-specific robotic designs and for clinicians to use task-oriented, individualized protocols to boost neuroplasticity and functional recovery. Consistent with prior work, pairing robotics with VR appears to enhance engagement and motor learning. Poor descriptions of devices and protocols remain a major limitation.	Low
Vezér et al., 2024 [39]	To examine the effect of RAGT on gait function and on temporospatial gait parameters in children with CP.	318	13	RAGT	The evidence for robotic-assisted gait training (RAGT) is mixed. While individual studies found gains in postural stability (GMFM-D), walking ability (GMFM-E), step length and gait velocity, pooled or controlled comparisons typically show no clear superiority of RAGT compared to dose-matched treadmill training or conventional therapy. Cadence and many other gait parameters usually remain unchanged. Major constraints are high heterogeneity, small sample sizes, risk of bias (often unblinded), variable training protocols, and lack of long-term follow-up.	Moderate
Volpini et al., 2022 [23]	To evaluate the short-term and long-term effects of RAGT on walking distance, gait speed, and functionality in individuals with CP.	77	7	RAGT	The review reports that robotic-assisted gait training yields large clinical improvements in gait speed and gross motor function (GMFM-D/E) and significantly increases walking endurance (6 MWT), with many gains persisting long term. However, statistical analyses sometimes fail to show significance (reflecting small samples and variability). Heterogeneous protocols, limited follow-up, and poor control of post-trial activities weaken conclusions. RAGT appears to be valuable as an adjunct but needs robust trials to confirm optimal dosing and durability.	High
Vova et al., 2019 [4]	To explore the efficacy of two modalities, FES and exoskeletons in gait training to improve motor function and improve gait efficiency in pediatric individuals with CP.	208	14	Lockomat, CPW, HAL, exoskeleton.	Across the reviewed studies, exoskeletons improved gait speed, endurance, gross motor function (GMFM D/E), hip/knee kinematics and reduced crouch gait. These effects were strongest when users were actively engaged and training included task variability. Functional Electrical Stimulation, both for ambulation and cycling, improved gait kinematics, ankle dorsiflexion strength, reduced spasticity and increased walking speed, balance and muscle activation. However, most studies had small samples, few controls, variable protocols and short follow-up, so larger standardized trials are needed.	Low
Wang et al., 2023 [24]	To evaluate the effectiveness of RAGT in improving lower extremity function in patients with CP and compare the efficacy between different robotic systems.	654	14	RAGT: LokoHelp, Lokomat, 3DCaLT, GTI	This review finds that robotic-assisted gait training produces meaningful gains in standing and walking function (GMFM D/E), postural balance (BBS) and walking endurance (6 MWT) compared with conventional rehab, with LokoHelp and Lokomat leading rankings and 3DCaLT showing little effect. Statistical improvements in gait speed were generally absent despite some clinically meaningful changes. Interpretation is constrained by small samples, lack of blinding, wide variability in age/CP severity, and inconsistent training frequency/duration.	Moderate

## Data Availability

No new data were created or analyzed in this study. Data sharing is not applicable to this article.
